# Full closed loop open‐source algorithm performance comparison in pigs with diabetes

**DOI:** 10.1002/ctm2.387

**Published:** 2021-05-01

**Authors:** Rayhan A. Lal, Caitlin L. Maikawa, Dana Lewis, Sam W. Baker, Anton A. A. Smith, Gillie A. Roth, Emily C. Gale, Lyndsay M. Stapleton, Joseph L. Mann, Anthony C. Yu, Santiago Correa, Abigail K. Grosskopf, Celine S. Liong, Catherine M. Meis, Doreen Chan, Joseph P. Garner, David M. Maahs, Bruce A. Buckingham, Eric A. Appel

**Affiliations:** ^1^ Division of Endocrinology Department of Medicine Stanford University Stanford California USA; ^2^ Division of Endocrinology Department of Pediatrics Stanford University Stanford California USA; ^3^ Stanford Diabetes Research Center Stanford University Stanford California USA; ^4^ Department of Bioengineering Stanford University Stanford California USA; ^5^ OpenAPS Seattle Washington USA; ^6^ Department of Comparative Medicine Stanford University Stanford California USA; ^7^ Department of Materials Science & Engineering Stanford University Stanford California USA; ^8^ Department of Biochemistry Stanford University Stanford California USA; ^9^ Department of Chemical Engineering Stanford University Stanford California USA; ^10^ Department of Chemistry Stanford University Stanford California USA; ^11^ Department of Psychiatry and Behavioral Sciences Stanford University Stanford California USA

**Keywords:** automated insulin delivery, diabetes, open‐source closed loop

## Abstract

Understanding how automated insulin delivery (AID) algorithm features impact glucose control under full closed loop delivery represents a critical step toward reducing patient burden by eliminating the need for carbohydrate entries at mealtimes. Here, we use a pig model of diabetes to compare AndroidAPS and Loop open‐source AID systems without meal announcements. Overall time‐in‐range (70–180 mg/dl) for AndroidAPS was 58% ± 5%, while time‐in‐range for Loop was 35% ± 5%. The effect of the algorithms on time‐in‐range differed between meals and overnight. During the overnight monitoring period, pigs had an average time‐in‐range of 90% ± 7% when on AndroidAPS compared to 22% ± 8% on Loop. Time‐in‐hypoglycemia also differed significantly during the lunch meal, whereby pigs running AndroidAPS spent an average of 1.4% (+0.4/−0.8)% in hypoglycemia compared to 10% (+3/−6)% for those using Loop. As algorithm design for closed loop systems continues to develop, the strategies employed in the OpenAPS algorithm (known as oref1) as implemented in AndroidAPS for unannounced meals may result in a better overall control for full closed loop systems.

## Graphical abstract

SHORT ABSTRACT: Understanding how algorithm features impact glucose control after unannounced meals is a critical step toward full closed loop insulin delivery and reduced patient burden. For unannounced meals in a full closed loop system, and with faster insulin pharmacokinetics inherent to pigs, time‐in‐range was greater with open‐source algorithm AndroidAPS than Loop in pigs with diabetes.

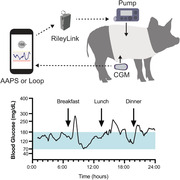

## 1 INTRODUCTION

Type 1 diabetes management is highly burdensome, requiring regular monitoring of blood glucose levels and careful insulin delivery. Accurate insulin dosing is critical to maintain glucose levels within the normal glycemic range and to prevent acutely dangerous hypoglycemic episodes as well as the long‐term consequences of hyperglycemia.^1^, ^2^, ^3^ The result is that individuals with type 1 diabetes must carefully deliver insulin in relationship to their meals and exercise to manage their blood glucose.

The recent advent of accurate continuous glucose monitors (CGM) has allowed the development of automated insulin delivery through continuous subcutaneous insulin infusion pumps.^1^, ^2^, ^4^ Automated insulin delivery systems have been associated with improved glycemic outcomes and reduced patient burden.^4^, ^5^ In September 2016, the FDA approved the first hybrid closed loop (HCL) device, the MiniMed 670G (Medtronic, Dublin, Ireland).^6^, ^7^ In December 2019, a second HCL system by Tandem Diabetes Care (San Diego, CA) dubbed Control‐IQ received FDA approval.^8^ Both devices use glucose measurements from a CGM to alter basal insulin delivery from an insulin pump through software running on an embedded microcontroller in the pump. Unfortunately, these are not fully automatic systems and users are still required to enter the quantity of carbohydrates they plan to eat into the pump for mealtime insulin boluses.^7^ A full closed loop system would enable entirely autonomous insulin delivery without user input, but these systems have yet to be successfully implemented commercially primarily due to a lack of efficient control in the post prandial period.^9^ Improvements in meal‐detection algorithms may present an avenue for improvement in completely autonomous control.^9^

**FIGURE 1:**
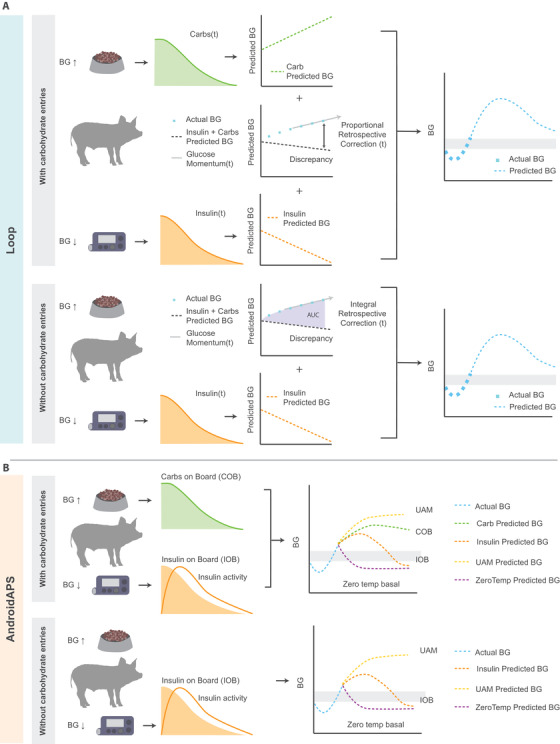
Algorithm schematic. Open‐source automated insulin delivery systems AndroidAPS and Loop have been designed to incorporate mealtime carbohydrate entry into blood glucose (BG) predictions, and thus control of insulin delivery. However, a system that does not require meal announcements needs to be able to respond to unannounced mealtime glucose excursions to maintain glucose within a target range. (A) Loop algorithm constructs a single glucose prediction based on the sum of four components: (i) carbohydrates on board, (ii) insulin on board, (iii) glucose momentum, and (iv) retrospective correction. Retrospective correction adjusts the predicted glucose level by measuring the discrepancy between the predicted glucose and the actual glucose levels. (B) AndroidAPS/OpenAPS algorithm constructs four separate prediction curves: (i) insulin predicted glucose, (ii) carbohydrate predicted glucose, (iii) unannounced meal (UAM) predicted glucose, and (iv) zero‐temporary basal insulin predicted glucose. The AndroidAPS/OpenAPS algorithm then aims for the minimum predicted glucose value to still fall within the target range

Open‐source algorithms for closed loop or automated insulin delivery (AID) predate commercial availability of such systems and have been developed by members of the diabetes community.^10^, ^11^, ^12^, ^13^ The three most common systems include OpenAPS, Loop, and AndroidAPS.^14^, ^15^ Each has an emphasis on safety but are unregulated, as they are user‐designed and user‐driven. As compared to commercial systems they offer significant transparency and personalization.^10^ Without conventional regulatory oversight, design changes can occur more rapidly. The systems use models to predict future glucose and then alter insulin delivery to optimize glucose to a target range specified by the user (Figure 1).

While these open‐source AID systems, like commercial systems, originally functioned most optimally with meal input from the user, efforts have been made to provide full closed loop control. The most commonly used algorithm with advanced features for full closed loop control is the OpenAPS algorithm known as “oref1” that is also used in AndroidAPS. The current heuristic algorithm of oref1 generates multiple future blood glucose predictions based on (i) action of approximated insulin remaining in the body; (ii) the scenario in which all carbohydrate intake ceases, and the system stops insulin delivery; (iii) action of approximated carbohydrates remaining in the body; and (iv) unannounced meal absorption. The predictions are then combined to estimate the lowest predicted glucose, and insulin delivery is adjusted to ensure that the local minima remains within a prespecified target. The user, similar to standard insulin pump therapy, inputs their personal basal rate, insulin‐to‐carbohydrate ratio (ICR), insulin sensitivity factor (ISF), and curve of insulin pharmacodynamics. OpenAPS and AndroidAPS also enable automated insulin coverage of meals without carbohydrate announcement via the “unannounced meal” feature, whereby meals are anticipated based on available data suggesting an otherwise unanticipated glycemic excursion. These unannounced meals may then be acted upon by a supermicrobolus (SMB) wherein tiny boluses are delivered to more rapidly affect rising glucose levels. Other features, not specific to unannounced meals, such as “auto‐sensitivity” respond to glucose fluctuations beyond the scope of predictions. These features used in combination enable some OpenAPS and AndroidAPS users to utilize the open‐source system in a full closed loop mode.

The other open‐source AID system, Loop, uses a different algorithm that employs coincidence point control (model predictive control),^16^ and generates a single future prediction based on insulin delivery history, carbohydrates entered, and other entered settings, including the basal rate, ICR, ISF, and curve of insulin pharmacodynamics. When used in humans, users enter not only carbohydrate counts but also an estimate of the absorption time of that particular meal or food. Loop uses two forms of short‐term adaptation called “glucose momentum” and “retrospective correction” to enact temporary basal rates to push the projected glucose toward a specified target range. Glucose momentum uses the 5‐min rates of change from the prior 20 min to influence future predictions, with most weight given to the most recent rate of change. In retrospective correction, glucose differing from the predicted value causes that difference to be added to the next prediction. An extension of this feature called “integral retrospective correction” takes not only the difference but also the accumulated differences into account for more rapid adaptation.

These open‐source algorithms have become increasingly popular amongst patients but are difficult to study in humans, outside of observational studies, due to regulatory restrictions.^10^, ^17^, ^18^ Work is just beginning on randomized controlled trials analyzing these systems in hybrid closed loop mode (where users are typically announcing meals); there are currently no head‐to‐head studies comparing automated insulin delivery system performance without meal announcement.^13^ In developing these algorithms further for use without meal announcements, it is important to understand the performance of each in clinical practice. In this study, we evaluate the algorithms used in OpenAPS/AndroidAPS and Loop, without meal announcements or user interactions, in a pig model of insulin‐deficient diabetes. The comparison of the algorithms in pigs, which have similar physiological glucose ranges but demonstrate faster insulin kinetics than humans, allows for algorithm performance to be evaluated as if using next‐generation ultra‐fast insulins that are in the pipeline but not yet available for use in humans.^19^, ^20^, ^21^ This is useful because it is hypothesized that ultrafast insulin kinetics may enable fully closed loop delivery without the requirement of additional therapeutics (glucagon or amylin) or sensors (accelerometers). Exploiting the faster insulin kinetics in pigs allows us to understand the important features of full closed loop algorithm design in the context of future ultra‐fast insulins. The quality of closed loop control was evaluated primarily by time‐in‐range (TIR) of 70–180 mg/dl and number of hypoglycemic events. We hypothesized that the unannounced meal and SMB features of the oref1 algorithm used in OpenAPS and AndroidAPS would result in superior closed loop control for unannounced meals as compared to Loop.

## 2 MATERIALS AND METHODS

### 2.1 Study design

This was a prospective animal study that examined the differences in performance between two open‐source automated insulin delivery system algorithms. The algorithms most commonly used are: oref1, used in OpenAPS and AndroidAPS, and the Loop algorithm. Six female Yorkshire pigs with streptozotocin‐induced diabetes were used in this study. Pigs were set up with the closed loop algorithm prior to the monitoring period. Algorithms remained constant for at least 1 day and the AndroidAPS algorithm preceded the Loop algorithm in all cases. Since each algorithm approaches communication with the RileyLink (and thus the pump) differently, studying a single algorithm at a time reduced the risk of unforeseen challenges where communication styles conflicted. AndroidAPS uses a polite strategy to coordinate and “take turns” querying the RileyLink when multiple rigs are running. In contrast, Loop constantly queries the RileyLink and when running Loop we were limited to three Loop rigs at a time. As described in the Supporting Methods, the current study was performed following a month‐long pharmacokinetic study in the same cohort of pigs with diabetes. There was a washout period of 12 h or more between the pharmacokinetic studies and the start of closed loop studies. All pharmacokinetic studies were performed with rapid‐acting insulins that would be cleared well within this period. The pigs were studied in the context of closed loop control over a 20‐day period, however due to technical difficulties with devices, monitoring periods were not completed on all days. Table S1 shows the dates of each individual monitoring period by meal and by pig. Reasons that monitoring periods were not completed included: (i) loss of signal from CGM, (ii) detachment of CGM, or (iii) detachment of infusion set during the monitoring period. It should be noted that from August 11 to 15, 2019 pigs were switched to AndroidAPS with a novel insulin formulation, and thus data from that period were not included in this study and from August 21 to 24, 2019 pigs 2, 3, 5, and 6 participated in pharmacokinetic experiments for a novel insulin formulation and were not participating in closed loop studies. Total monitoring periods across all pigs were 69 for AndroidAPS and 47 for Loop. However, these totals include repeated observations in the same subject (pig) and conditions (meal and algorithm) (Table S2). Total independent observations were 24 for AndroidAPS and 23 for Loop (six pigs, four meals, two algorithms). During experiments where the Loop algorithm was used only two to three pigs could be tested at one time to prevent radio interference between competing rigs, which contributed to the lower monitoring period count for the Loop algorithm and greater spacing between recordings. This study was a secondary study and thus sample size was not based on power analysis specific to this study. However, Mead's resource equation (based on curves of diminishing returns) suggests that total error degrees of freedom (DF) should fall between 10 and 20 in order to detect worthwhile effect sizes.^22^ The denominator DF (i.e., error DF) is reported in Section 3 alongside all *F* ratios (*F*_numerator DF, denominator DF_).

TIR of 70–180 mg/dl was defined as the proportion of 5‐min intervals that were in the euglycemic range out of the total number of intervals during each mealtime monitoring period. Time‐in‐hypoglycemia was defined as the proportion of 5‐min intervals where glucose was <70 mg/dl.

### 2.2 Streptozotocin‐induced diabetes in swine

Female Yorkshire pigs (Pork Power) were used for all experiments. Animal studies were performed in accordance with the guidelines for the care and use of laboratory animals and all protocols were approved by the Stanford Institutional Animal Care and Use Committee. Insulin‐deficient diabetes was induced in pigs (25–30 kg) using streptozotocin (STZ) (MedChemExpress), as previously reported.^19^, ^20^ STZ was infused intravenously at a dose of 125 mg/kg and animals were monitored for 24 h. Food and administration of 5% dextrose solution was given as needed to prevent hypoglycemia. Diabetes was defined as fasting blood glucose greater than 300 mg/dl. For closed loop studies, pigs were set up with a continuous glucose monitor (Dexcom G6) applied to the pig's lower side toward their rear flank (Figure S5). The insulin pump was placed in the pocket of a pig jacket (Lomir Biomedical) and cannula was inserted subcutaneously in a similar location to the CGM either on the opposite side of the pig, or more than 3 inches from the CGM if on the same side. The Rileylink, a communication bridge device used to communicate between the insulin pump and the mobile phone, was secured within range of both the phone and insulin pump in the pig's housing.

### 2.3 Determination of insulin needs parameters in pigs with diabetes

As with type 1 diabetes in humans, STZ‐induced diabetes manifests with different insulin requirements in each pig. In response, we individualized dosing regimens and customized closed loop setups for each animal. To customize closed loop setups for each pig, individual dosing parameters were derived without closed loop control (Table 1). First, basal insulin requirements were determined by titration to maintain nocturnal glucose within 30 mg/dl of starting glucose values. Basal rates ranged between 0.10 and 0.30 units/h. During these titrations, pigs wore CGMs and insulin pumps and the basal rate was iteratively adjusted through trial and error until glucose levels remained stable overnight. Upon establishing this basal rate, ISF was established by measuring glucose response to 1 unit of insulin delivered intravenously simultaneously with subcutaneous basal insulin infusion in the unfed state. ISF was equal to the change in glucose after insulin administration (glucose typically took ∼90 min to stabilize). ISF ranged from 110 to 167 mg/dl/unit. ICR was determined by titrating mealtime insulin bolus to maintain pre‐ and post‐prandial glucose following a 400 g meal of pig chow (132 g carbohydrates) with titrated basal in the absence of hyperglycemia correction. Without carbohydrate announcements neither system utilizes ICR, and thus ICR was not a critical parameter in the context of this study.

**TABLE 1:** Individual insulin dosing parameters

Pig	Basal rate (units/h)	ISF (mg/dl/unit insulin)	ICR (g carb/unit insulin)
1	0.15	110	49
2	0.20	167	90
3	0.10	154	68
4	0.30	158	59
5	0.20	160	69
6	0.25	132	58

### 2.4 Insulin pharmacokinetics and pharmacodynamics in pigs with diabetes

**FIGURE 2:**
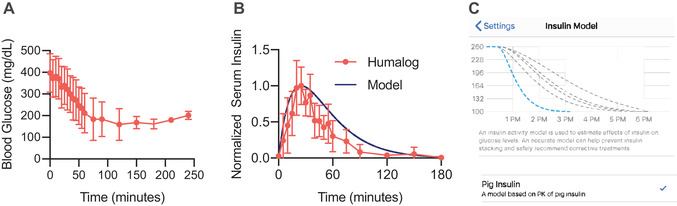
Pharmacokinetics and pharmacodynamics used for insulin model. Fasted pigs with diabetes were injected subcutaneously with 4 U of Humalog. (A) Blood glucose measurements in fasted pigs. (B) Insulin (lispro) pharmacokinetics and corresponding model fit. (C) Modeled insulin pharmacodynamics from within Loop.

Insulin pharmacokinetics are more rapid in pigs than in humans.^19^, ^20^, ^29^ Thus, it was necessary to construct models for insulin action to be used in both open‐source systems, which model insulin action as an exponential curve. In AndroidAPS, the parameters defining the curve are time‐to‐peak action (TPA) and total duration of insulin action (DIA).^23^ Loop uses time‐to‐onset, TPA, and DIA.^15^ The pig pharmacokinetic curves for use in open‐source algorithms were determined from data obtained during a pilot study in pigs (*n* = 6). This experimental pharmacokinetic curve is consistent with pharmacokinetics observed in the pigs used in this study.^20^ Pigs were kept on fast 4–6 h and then injected subcutaneously with a 4 U dose of Humalog (100 U/ml, Eli Lilly). Before injection, baseline blood was sampled from an intravenous catheter line and measured using a handheld glucose monitor (Bayer Contour Next). After injection, blood was sampled from the intravenous catheter line every 5 min for the first 60 min, then every 30 min up to 4 h. Blood was collected in K_2_EDTA plasma tubes (Greiner‐BioOne) for analysis with ELISA. Plasma lispro concentrations were quantified using an Insulin Lispro ELISA kit (Mercodia). Pharmacodynamics were approximated by blood glucose measurements in a stable fasting state after 2–3 h (Figure 2A). Insulin pharmacokinetics demonstrated peak serum concentrations within 20–25 min and near complete exposure duration of 2 h (Figure 2B). Our pig insulin model was developed with initial glucose lowering effect at 15 min (used only for Loop), peak action of 25 min, and DIA of 3 h to prevent insulin stacking. Figure 2C shows the insulin model setting within Loop. Initial pilot studies with a DIA of 2 h showed increased hypoglycemia from mis‐approximation of insulin clearance.

### 2.5 AndroidAPS

AndroidAPS is an android‐based open‐source automated insulin delivery system that utilizes the same algorithm from OpenAPS. The OpenAPS algorithm, known as oref0 or as oref1 when implemented with advanced features, determines insulin dosing based on a number of scenarios that it forecasts with different types of predictions, which are then blended to determine appropriate insulin adjustments.^23^ Two of these scenarios, “eventual” (eventualBG) and “IOB‐based” (IOBpredBGs), attempt to predict BGs in situations without (much) carbohydrate absorption. Another scenario, “zero‐temp” (ZTpredBGs), attempts to predict the “worst likely case” if observed carbohydrate absorption suddenly ceases and if a zero‐temp were applied until BG begins rising at/above target. The final two scenarios, “COB” (COBpredBGs) and “unannounced meal” (UAM) (UAMpredBGs), attempt to predict how long an observed BG rise will continue, to dose appropriately for announced and unannounced meals, and for anything else that causes a sustained rise in BG.

When no carbohydrate announcements are available, or when announced carbohydrates are mostly absorbed and COB‐based predictions are less reliable, it is also possible to predict that observed deviations would gradually return to zero over some period (a “deviation” term is calculated to represent how much BG is currently rising or falling relative to what it should be doing based solely on insulin activity).

Once deviations have peaked and are decreasing at a reasonable rate, the UAM calculations assume that the deviations will continue to decrease at the same rate until they reach zero. If they are decreasing, but too slowly, it assumes they will decrease linearly to zero over 3 h. If deviations are still increasing, it assumes they will peak immediately and start decreasing at one‐third of the rate they increased from their recent minimum.

After oref0 generates all relevant predictions, it blends and combines them to produce estimates of the lowest predicted BGs likely to be observed over the timeframe relevant for dosing, calculates how much insulin is required (insulinReq) to bring the minimum predicted BG down toward the target, and then uses the insulinReq to calculate an appropriate microbolus or temp basal. If no carbohydrate announcement is present, minPredBG is generally set to the maximum of minIOBPredBG, the lowest IOBpredBG (starting 90 min in the future), and minZTUAMPredBG, which is the average of the lowest UAMpredBG (starting ∼60 min in the future, minUAMPredBG) and the lowest ZTpredBG (starting immediately, minZTGuardBG).

The insulin recommended to be dosed (insulinReq) is then set to the difference between the minPredBG and target BG, divided by ISF. During each loop (calculation), half of the insulinReq is delivered as a microbolus, when not limited by other safety settings, and on each subsequent loop the minPredBG is recalculated to calculate a new insulinReq and microbolus.^24^ This implementation of oref1 in AndroidAPS with unannounced meals and SMB has been tested in other studies, primarily in real‐world usage with meal announcements, and also studied in silico.^25^, ^26^, ^27^

AndroidAPS code (https://github.com/MilosKozak/AndroidAPS) and documentation (https://androidaps.readthedocs.io/en/latest/EN/) are available online.

### 2.6 Loop

Loop is another open‐source automated insulin delivery system that uses a different algorithm that employs model predictive control to forecast future glucose and then delivers insulin and keeps glucose within a user set target. A suspend threshold stops all insulin delivery when the actual or predicted glucose is below a prespecified value. In normal use, the effect of delivered insulin and entered carbohydrates is modeled with additional short‐term adaptation performed by “glucose momentum” and “retrospective correction.” With unannounced meals, the system is unable to model the effect of carbohydrates. Instead, these excursions are perceived as deviations from prior predictions and thus must be acted upon by the short‐term adaptations. The integral retrospective correction feature is hypothesized to improve glucose control under unannounced meal conditions compared to standard retrospective correction.^28^ Further information about the standard retrospective correction factor originally used by Loop can be found in “Loop: Integral retrospective correction.”^28^ Loop code for the main branch (https://github.com/LoopKit/Loop), integral retrospective correction branch (https://github.com/dm61/Loop/tree/integral‐retrospective‐correction), and documentation (https://loopkit.github.io/loopdocs/) are available online.

### 2.7 Closed loop challenge

Pigs with streptozotocin‐induced insulin‐deficient diabetes were started on the open‐source closed loop systems AndroidAPS (Build: MDT_0.11‐66‐g264bbdee2‐2019.08.02‐23:07, running the oref1 algorithm with SMB and UAM enabled) and Loop (Build: 1.10.0dev‐jojo, using the Loop algorithm with integral retrospective correction enabled). Despite identical algorithm settings for each pig, only two of the six pigs (Pigs 4 and 5) received SMB and basal rate modulation from AndoidAPS. Single SMB amounts are limited by several factors. The largest a single SMB bolus can be is the smallest value of (1) 30 min of the current regular basal rate (as adjusted by autotune/autosens); (2) half of the insulin required amount; or (3) the remaining portion of your maxIOB setting in preferences. The relatively low insulin requirements may have limited the delivery of microboluses in the remaining pigs. For the remaining pigs, only temporary basal rates were utilized to control glucose. Pigs were fed three meals per day at approximately 7:30 a.m., 1:30 p.m., and 7:00 p.m. each day. Breakfast consisted of applesauce with added sugar (66 g carbohydrates) to mimic a fast‐absorbing simple carbohydrate meal. Lunch and dinner were standard pig chow (400 g Teklad Miniswine Diet 8753; 132 g carbohydrates).

The post‐prandial monitoring period for each meal was considered to be the 6 h following breakfast, or the 5 h following lunch or dinner. Overnight monitoring was defined as the 6 h between 1:00 a.m. and 7:00 a.m. Monitoring periods where CGMs stopped working for longer than 1 h were excluded. Periods where CGMs or infusion sets fell off were also excluded. No software malfunctions for AndroidAPS or Loop occurred that required data exclusion. TIR was calculated for each post‐prandial or overnight monitoring period.

During the closed loop challenge, corrective carbohydrates were given when two methods of glucose measurement (e.g., ear prick, iv blood draw, or CGM) were <55 mg/dl or if the CGM alone reported glucose <40 mg/dl. Here, we use administration of corrective carbohydrates as a metric for severe hypoglycemic event. Glucose level of 55 mg/dl was used as the threshold for corrective carbohydrate administration as it is the default “Urgent Low Soon” alarm setting for the Dexcom G6 in the Dexcom app. However, observationally the Dexcom G6 often slightly underestimated glucose levels (a reasonable safety feature) compared to glucose measurements taken using a handheld monitor from an ear prick or venous catheter line blood sample. To avoid administering corrective carbohydrates unnecessarily, we set the requirements that two measurements had to indicate that blood glucose was <55 mg/dl. Typically, the “Urgent Low Soon” alarm would go off and then the pig's blood glucose would be taken by a secondary method to confirm whether corrective carbohydrates were required (two measurements <55 mg/dl). Alternatively, we set a threshold of CGM reading alone of <40 mg/dl could also warrant administration of corrective carbohydrates. This limit was set because some pigs had a strong aversion to ear pricks by the end of the study and always taking two measurements was not possible. The lower limit was set based on observation that typically a CGM value alone just <55 mg/dl did not correspond to severe hypoglycemia by other methods.

### 2.8 Statistics

For statistical analysis time‐in‐hypoglycemia required additional transformation using the natural logarithm to meet assumptions of homoscedasticity. No transform was performed on the TIR data.

All analyses were performed in JMP Pro 14 or SAS Version 9.4. As each pig acted as its own control, we used repeated measures. To test if glucose TIR or time‐in‐hypoglycemia differed between algorithms, we used a restricted maximum likelihood (REML) repeated measures mixed model. Pig was included as a random effect subject. Algorithm and meal were included as within‐subject fixed effects. The interaction between algorithm and meal (algorithm*meal) tested whether the algorithms differed in performance for different meals. Post hoc tests of significant interactions were performed as Bonferroni corrected planned contrasts, where the effect of algorithm was tested for each meal, and significance was set at *p* < .0125.

Severe hypoglycemic events were identified as when two methods of glucose measurement (e.g., ear prick, iv blood draw, or CGM) were <55 mg/dl or if the CGM alone reported glucose <40 mg/dl. For each session, the pig was scored as experiencing a severe hypoglycemic event or not. As each pig acted as its own control, we used repeated measures, and as each trial was a simple yes or no, we used logistic regression. To accommodate repeated measures appropriately, we implemented the logistic regression as a GEE within a generalized linear model with a logistic link function and a binomial error distribution in PROC GENMOD (SAS Version 9.4). The effect of algorithm was tested with likelihood ratios. Least‐squares means and standard error (SE) (i.e., mean probability corrected for pig) were calculated and plotted or reported in the text.

To test if insulin requirements in the 5 h following the start of monitoring periods differed between algorithms, we used REML repeated measures mixed model. Pig was included as a random effect subject. Algorithm and meals were included as within‐subject fixed effects. The interaction between algorithm and meal (algorithm*meal) tested whether the algorithms differed in performance for different meals.

## 3 RESULTS

### 3.1 Closed loop challenge in swine with diabetes

**FIGURE 3:**
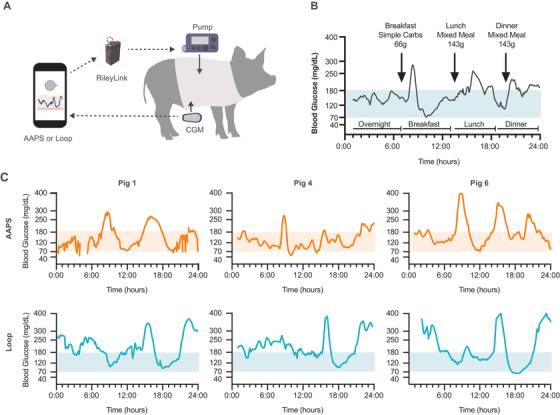
Glucose traces during closed loop challenges. Pigs with streptozotocin‐induced insulin‐deficient diabetes underwent head‐to‐head comparison of do‐it‐yourself open‐source closed loop systems AndroidAPS and Loop. (A) Pigs wear Dexcom G6 continuous glucose monitors (CGM) and compatible Medtronic pumps that connect to either an android phone (AndroidAPS) or iPhone (Loop) via a RileyLink. Systems are setup for full closed loop (no meal announcements). (B) Glucose levels are monitored overnight and after three meal challenges per day. (C) Sample full‐day traces for both AndroidAPS and Loop for pigs 1, 4, and 6. Time‐in‐range (euglycemia) was defined as the time where CGM measured glucose was 70–180 mg/dl

**FIGURE 4:**
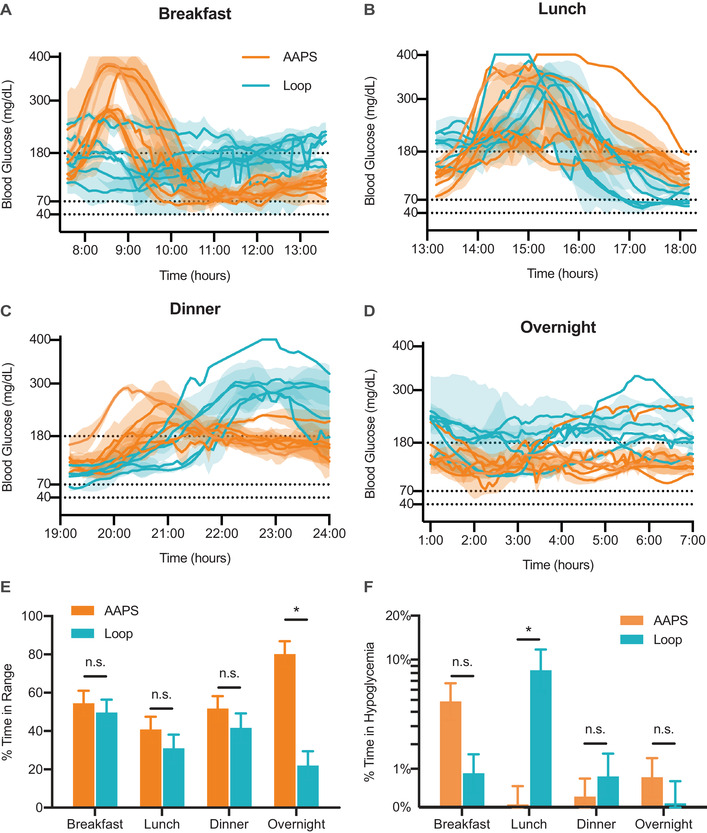
Time spent in range and hypoglycemia during closed loop challenges. Average traces (mean ± SE) for each pig on AndroidAPS or Loop for (A) breakfast, (B) lunch, (C) dinner, (D) overnight. Time‐in‐range (TIR) was defined as time where CGM measured glucose between 70 and 180 mg/dl and hypoglycemia was defined <70 mg/dl. (E) TIR is reported as a percentage of the total time during the monitoring period: breakfast (6 h), lunch (5 h), dinner (5 h), or overnight (6 h) for AndroidAPS and Loop algorithms (F), time‐in‐hypoglycemia is reported as a percentage of the total time during the monitoring period (6 h for overnight and breakfast; 5 h for lunch and dinner. Data are shown as log‐transformed least squares mean ± SE with back‐transformed axis labels. (A–D) Glucose curves are shifted on the *x*‐axis to align the start times of the monitoring periods. (E and F) Each pig was monitored for each meal at least once for each algorithm. Data are reported as least squares mean ± SE. Statistical significance was determined by restricted maximum likelihood (REML) repeated measures mixed model. Bonferroni post hoc tests were performed on individual meal test slices and significance (*) and alpha was adjusted to account for multiple comparisons (alpha = .0125)

To test the algorithms, pigs were set up on either AndroidAPS or Loop closed loop systems (Figure 1, Figures S1 and S2). During the study, the pigs were monitored over four “meal” monitoring periods: breakfast, lunch, dinner, and overnight (no food was given during the overnight “meal”) (Figure 3A,B). Sample full day monitoring glucose profiles for three pigs comparing AndroidAPS and Loop are shown in Figure 3C. Average glucose levels for each individual pig are shown broken down by meal in Figure 4A–D (see Figures S3–S6 for individual curves). Overall mean ± SE TIR for AndroidAPS was 58% ± 5%, while TIR for Loop was 35% ± 5%. On average, TIR differed between algorithms (*F*_1,5.43 _= 15.16; *p*‐value = .0098) and, between meals (*F*_3,17.77 _= 3.786; *p* = .0291), but in fact the effect of algorithm differed between meal periods (*F*_3,15.97 _= 22.09; *p* < .0001) such that the algorithms only differed significantly during the overnight monitoring period (Figure 4, Table S3). Within the overnight monitoring period, pigs had an average TIR of 90% ± 7% when on the AndroidAPS algorithm compared to 22% ± 8% when on Loop (post hoc planned contrast: *F*_1,16.09 _= 67.34; *p* < .0001). Observation of individual glucose curves indicates that TIR differences between algorithms during the overnight period appear to be a result of prolonged post‐prandial hyperglycemia after dinner in pigs using Loop. Algorithm did not have an effect on the amount of insulin delivered (Figure S7).

**FIGURE 5:**
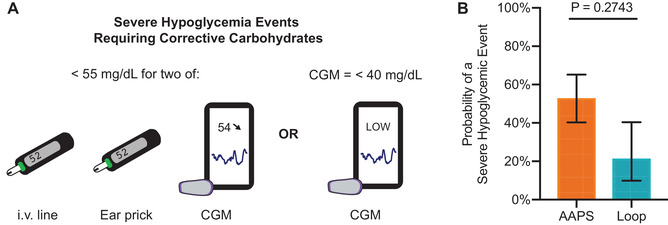
Hypoglycemic events requiring corrective carbohydrates. When corrective carbohydrates were required, pigs received half a jumbo marshmallow (10 g sugar). Corrective carbohydrates were only necessary during breakfast monitoring periods for both algorithms. (A) Severe hypoglycemic events that required corrective carbohydrates were defined as (i) when two methods of glucose measurement were <55 mg/dl (ear prick, iv blood draw, or CGM), or (ii) when the CGM alone reported glucose <40 mg/dl. (B) Probability of severe hypoglycemic events during the breakfast monitoring period for each algorithm. Each breakfast monitoring period was evaluated as having one of two outcomes: (i) no intervention was necessary, or (ii) corrective carbohydrates were given. Data shown are back‐transformed from log‐transformed least squares mean ± SE. Each pig received each algorithm at least once (*n* = 6 pigs). Statistical significance was determined using logistic regression as a GEE within a generalized linear model. Each pig acted as its own control and was included as repeated measures

The characterization of hypoglycemia during closed loop challenges were split into two metrics: (i) time spent in hypoglycemia (defined as blood glucose <70 mg/dl); and (ii) severe hypoglycemic events requiring corrective carbohydrates (Figures 4 and 5). On average, time‐in‐hypoglycemia did not differ between algorithms (*F*_1,4.515 _= 0.6394; *p* = .4639), but differed between meals (*F*_3,16.1 _= 5.7022; *p* = .0074). However, the effect of algorithm differed with meal (*F*_3,14.21 _= 9.9186; *p* = .0009) such that the algorithms only differed significantly during the lunch meal. Pigs running AndroidAPS spent an average of 1.4% (SE = +0.4/−0.8)% in hypoglycemia during the lunch monitoring period compared to 10% (SE = +3/−6)% when using Loop (post hoc planned contrast: *F*_1,19.09 _= 19.78; *p* = .0003).

Corrective carbohydrates were given when two methods of glucose measurement (e.g., ear prick, iv blood draw, or CGM) were <55 mg/dl or if the CGM alone reported glucose <40 mg/dl. Corrective carbohydrates were only necessary during the rapid carbohydrate breakfast monitoring periods (Figure 4B), and no corrective carbohydrates were needed during other monitoring periods. The choice of algorithm did not significantly affect the probability of a severe hypoglycemic event requiring carbohydrates (LR Chi sq = 1.20; DF = 1; *p* = .2743).

## 4 DISCUSSION

In this study, we compare the open‐source algorithms used in common AID systems (oref1 in OpenAPS and AndroidAPS, and Loop) in a full closed loop challenge in pigs with type 1‐like diabetes. Breakfast was the only monitoring period where incidents of corrective carbohydrates occurred for either algorithm. These observations suggest that both algorithms over‐deliver insulin in response to fast‐absorbing carbohydrates but can adjust delivery to prevent hypoglycemia in response to mixed meals. From observing individual glucose profiles, it appears that Loop may have had difficulty adapting to differences in meal composition. Loop performs similarly to AndroidAPS at breakfast but causes fewer incidents of hypoglycemia requiring corrective carbohydrates. In the lunch post‐prandial period, the Loop algorithm led to increased time spent in hypoglycemia compared to the AndroidAPS algorithm. During the dinner post‐prandial period, the Loop algorithm exhibited prolonged hyperglycemia, which extended into the overnight monitoring period and translated to the observed decrease in overnight TIR. After the unannounced rapid rise in glucose at breakfast that continued at lunch, the system predicted a greater subsequent rise in glucose that increased aggressiveness of the insulin response, causing the observed hypoglycemia. As there is greater time duration between lunch and dinner, the retrospective correction abates and may even push the prediction downward, resulting in a more conservative insulin delivery and commensurate prolonged hyperglycemia. In contrast, AndroidAPS demonstrated consistent post‐prandial performance after lunch and dinner but showed a trend for increased hypoglycemia after breakfast.

These results suggest that Loop may have difficulty adapting sufficiently to unannounced meals, which are absorbed in a dynamic and variable fashion. We postulate that this observed behavior arises due to the selected time constants employed for this short‐term adaptation, which were selected for human use and are constant for both rising and falling glucose. The unannounced meal feature in AndroidAPS dynamically evaluates unanticipated upward deflections in glucose, and following peak deviation and sufficient fall, the algorithm predicts how long it will take for the unannounced meal to finish absorbing. Another difference between the systems is the choice of delivery method with boluses (Pigs 4 and 5 only) used occasionally in AndroidAPS versus basal modulation alone in Loop. Compared to boluses, basal modulation can still deliver relatively large quantity of insulin over a short period of time (up to 35 units/h). There may be a slight advantage to bolus delivery when connectivity is intermittent. Temporary basal rates are usually enacted for 30 min, whereas boluses are delivered completely once issued, so a disconnect may result in prolonged delivery of an altered basal rate. Thus, the AndroidAPS algorithm with SMBs and unannounced meal features may be better suited for the variability inherent to mixed meals than Loop.

In conclusion, we hope this study helps to inform the design and development of more effective algorithms for fully autonomous insulin delivery. Study in pigs presents an opportunity to explore algorithms in conjunction with faster insulin kinetics that are in the pipeline but not yet available for use in humans. A limitation of the study includes the frequent device failures (arising from sensor signal loss or devices falling off of the pigs) which meant that continuous closed loop monitoring over the course of an entire day was not always possible. Another limitation is that during AndroidAPS use, microboluses were only implemented for two of the pigs despite the application of identical settings. Further, the time constraints of this study, and differences in communications between rig set‐ups, meant that the algorithms were not randomized and the total number of observation periods with AndroidAPS exceeded the number of observation periods with Loop.

We have identified that current commercial algorithms are not yet optimized for fully autonomous control, and that it is important for future designs to be able to adapt to unannounced meals with variable absorption. Full closed loop has been attempted with newer ultra‐rapid insulin analogs alone^30^ but has been unable to achieve TIR target of >70%.^31^ Other attempts have required additional data (e.g., accelerometer for recognizing sleep^32^) or pharmaceuticals (e.g., amylin,^33^ glucagon^34^) to compensate for the limits of insulin‐only systems. We hope to identify whether open‐source algorithms could aid in efforts to achieve full closed loop without requiring additional pharmaceuticals/sensors. The open‐source algorithm of oref1, used in both OpenAPS and AndroidAPS, with unannounced meal feature and SMBs performs admirably under full closed loop conditions and should be considered by all when designing future algorithms. Moreover, with the advent of new biosensor technologies, closed loop control may become possible for other pharmacotherapy applications (e.g., immunosuppressants,^35^ pain management drugs,^36^ antiepileptic drugs,^37^ and anticoagulants).^35^ There is potential that our efforts to explore algorithm performance may potentially inform the development of systems for future applications of closed loop control of other molecules.

## CONFLICT OF INTEREST

Rayhan A. Lal has consulted for GlySens Incorporated, Abbott Diabetes Care and Biolinq, Capillary Biomedical, Morgan Stanley, and Tidepool. Of note, Dana Lewis is a developer and creator of the OpenAPS algorithm, which is used by both OpenAPS and AndroidAPS, but her work in the open‐source community is noncommercial and a nonfunded endeavor. Dana Lewis has not been paid for her work on the algorithm or for her contributions to this study or manuscript. No funding was provided by any of the open‐source AID projects. David M. Maahs has consulted for Abbott, Eli Lilly, the Helmsley Charitable Trust, Insulet, Novo Nordisk, and Sanofi. Bruce A. Buckingham has received research support from Medtronic, Tandem, Insulet, and Dexcom, and is on medical advisory boards for Convatec, Medtronic, Capillary Biomedical, Tidepool, and Tolerion. The remaining authors have no conflict of interest.

## AUTHOR CONTRIBUTIONS

Caitlin L. Maikawa, Rayhan A. Lal, and Eric A. Appel designed experiments and wrote the manuscript. Rayhan A. Lal assisted with set‐up of the DIY systems. Dana Lewis provided description of the oref1 algorithm and other technical sections and provided AndroidAPS code modifications to bypass the safety objectives sequences. Caitlin L. Maikawa, Anton Smith, L.Z, Gillie A. Roth, Lyndsay M. Stapleton, Emily C. Gale, Anthony C. Yu, Joseph L. Mann, Santiago Correa, Abigail K. Grosskopf, Catherine M. Meis, Doreen Chan, and Celine S. Liong performed experiments. Sam W. Baker performed surgeries for pigs and provided scientific input. Caitlin L. Maikawa, Rayhan A. Lal, and Joseph P. Garner analyzed the data. David M. Maahs, Bruce A. Buckingham, and Joseph P. Garner reviewed the manuscript and provided scientific input. Eric A. Appel served as principal investigator and reviewed and revised the manuscript. All the authors provided feedback and contributed to writing.

## Supporting information

Supporting Information

## Data Availability

The data supporting the results in this study are available within the Article and its Supporting Information. The broad range of raw datasets acquired and analyzed (or any subsets of it), which for reuse would require contextual metadata, are available from the corresponding author upon reasonable request.
