# Supplementary material for: Full closed loop open‐source algorithm performance comparison in pigs with diabetes
Source: Clin Transl Med. 2021 May 1;11(4):e387. doi: 10.1002/ctm2.387 (PMC8087942; doi:10.1002/ctm2.387)
Supplement: Supplementary file 1 — Supporting Information [file CTM2-11-e387-s001.pdf]

# Supplemental Information

## Full Closed-Loop Open Source Algorithm Performance Comparison in Pigs with Diabetes

Rayhan A. Lal[1,2,3+], Caitlin L. Maikawa[4+], Dana Lewis[5], Sam W. Baker[6], Anton A. A. Smith[7], Gillie A. Roth[4], Emily C. Gale[8], Lyndsay M. Stapleton[4], Joseph L. Mann[7], Anthony C. Yu[7], Santiago Correa[7], Abigail K. Grosskopf[9], Celine S. Liong[4], Catherine M. Meis[7], Doreen Chan[10], Joseph P. Garner[6,11], David M. Maahs[2,3], Bruce A. Buckingham[2,3], Eric A. Appel [2,3,4,7]

[+] These authors contributed equally to this work

[1] Division of Endocrinology, Department of Medicine, Stanford University, Stanford CA 94305, USA

[2] Division of Endocrinology, Department of Pediatrics, Stanford University, Stanford CA 94305, USA

[3] Stanford Diabetes Research Center, Stanford University, Stanford CA 94305, USA

[4] Department of Bioengineering, Stanford University, Stanford CA 94305, USA

[5] OpenAPS, Seattle WA, USA

[6] Department of Comparative Medicine, Stanford University, Stanford CA 94305, USA

[7] Department of Materials Science & Engineering, Stanford University, Stanford CA 94305, USA

[8] Department of Biochemistry, Stanford University, Stanford CA 94305, USA

[9] Department of Chemical Engineering, Stanford University, Stanford CA 94305, USA

[10] Department of Chemistry, Stanford University, Stanford CA 94305, USA

[11] Department of Psychiatry and Behavioral Sciences, Stanford University, Stanford CA 94305, USA

## SUPPLEMENTAL METHODS

### *Study Design*

This study was carried out as a secondary study in pigs with diabetes. The 6 pigs with diabetes used in this study participated in a primary study collecting pharmacokinetic data for novel insulin formulations, including an insulin/pramlintide co-formulation and an ultrafast insulin formulation. For these experiments, pigs were given an insulin bolus after an overnight fast or with a small meal and blood samples were taken over four hours. These pharmacokinetic studies took place over the first month after diabetes induction. Following the pharmacokinetic studies, closed-loop studies were performed where we observed an improvement in glucose control while running FCL with AndroidAPS compared to previous pilot data running FCL with Loop. We decided to investigate the differences between AndroidAPS and Loop running FCL head-to-head in our 6 pigs with diabetes and added FCL with Loop at the end of the study.

### *Comparison of pig and human mealtime absorption*

The gastrointestinal system of pigs is similar to that of humans, which should make pigs a good model for studying meal absorption[1,2]. Transit time through the stomach in pigs ranges from 0.8-1.3 hours compared to 0.15-2 hours in humans[2]. Similarly, small intestine transit time is 3-4 hours in pigs and 2-4 hours in humans[2]. That being said, pigs are still a model and there are differences between pigs and humans. Many studies report similar gastric emptying times between pigs and humans[3,4]. There is some disagreement on pig gastric emptying times in the literature, however it seems these larger differences appear to primarily occur in studies dosing oral drugs in caplet or tablet form rather than mixed/liquid meals.

Other differences in gastric emptying between humans and pigs can depend on a number of factors such as meal composition, volume, osmolarity, and viscosity of the meal [4]. This may account for the range of values reported in the literature. In this study we fed the pigs with a liquid meal of high in simple carbohydrates (applesauce), as well as standard pig chow representing a highly balanced meal to test algorithm performance under different meal conditions.

**Table S1: Log of successful monitoring periods**

| AndroidAPS | | | Loop | | |
|---|---|---|---|---|---|
| **Date (MM/DD/YY)** | **Meal** | **Pig** | **Date (MM/DD/YY)** | **Meal** | **Pig** |
| 8/6/19 | 4-Dinner | 3 | 8/17/19 | 2-Breakfast | 2 |
| 8/6/19 | 4-Dinner | 5 | 8/17/19 | 3-Lunch | 2 |
| 8/7/19 | 1-Overnight | 1 | 8/17/19 | 4-Dinner | 2 |
| 8/7/19 | 2-Breakfast | 1 | 8/17/19 | 2-Breakfast | 3 |
| 8/7/19 | 3-Lunch | 1 | 8/17/19 | 3-Lunch | 3 |
| 8/7/19 | 4-Dinner | 1 | 8/17/19 | 1-Overnight | 5 |
| 8/7/19 | 1-Overnight | 2 | 8/17/19 | 2-Breakfast | 5 |
| 8/7/19 | 2-Breakfast | 2 | 8/17/19 | 3-Lunch | 5 |
| 8/7/19 | 3-Lunch | 2 | 8/17/19 | 4-Dinner | 5 |
| 8/7/19 | 4-Dinner | 2 | 8/18/19 | 1-Overnight | 2 |
| 8/7/19 | 1-Overnight | 3 | 8/18/19 | 2-Breakfast | 2 |
| 8/7/19 | 3-Lunch | 4 | 8/18/19 | 3-Lunch | 2 |
| 8/7/19 | 4-Dinner | 4 | 8/18/19 | 1-Overnight | 3 |
| 8/7/19 | 1-Overnight | 5 | 8/18/19 | 2-Breakfast | 3 |
| 8/7/19 | 2-Breakfast | 5 | 8/19/19 | 4-Dinner | 2 |
| 8/7/19 | 3-Lunch | 5 | 8/20/19 | 1-Overnight | 1 |
| 8/7/19 | 4-Dinner | 5 | 8/20/19 | 2-Breakfast | 1 |
| 8/7/19 | 2-Breakfast | 6 | 8/20/19 | 3-Lunch | 1 |
| 8/7/19 | 3-Lunch | 6 | 8/20/19 | 4-Dinner | 1 |
| 8/7/19 | 4-Dinner | 6 | 8/20/19 | 2-Breakfast | 5 |
| 8/8/19 | 1-Overnight | 1 | 8/20/19 | 3-Lunch | 5 |
| 8/8/19 | 2-Breakfast | 1 | 8/22/19 | 2-Breakfast | 4 |
| 8/8/19 | 2-Breakfast | 3 | 8/22/19 | 3-Lunch | 4 |
| 8/8/19 | 3-Lunch | 3 | 8/22/19 | 4-Dinner | 4 |
| 8/8/19 | 4-Dinner | 3 | 8/23/19 | 2-Breakfast | 1 |
| 8/8/19 | 1-Overnight | 4 | 8/23/19 | 3-Lunch | 1 |
| 8/8/19 | 2-Breakfast | 4 | 8/23/19 | 4-Dinner | 1 |
| 8/8/19 | 3-Lunch | 4 | 8/23/19 | 1-Overnight | 4 |
| 8/8/19 | 4-Dinner | 4 | 8/23/19 | 2-Breakfast | 4 |
| 8/8/19 | 2-Breakfast | 5 | 8/23/19 | 3-Lunch | 4 |
| 8/8/19 | 1-Overnight | 6 | 8/23/19 | 4-Dinner | 4 |
| 8/8/19 | 2-Breakfast | 6 | 8/24/19 | 1-Overnight | 1 |
| 8/8/19 | 3-Lunch | 6 | 8/24/19 | 2-Breakfast | 1 |
| 8/8/19 | 4-Dinner | 6 | 8/24/19 | 3-Lunch | 1 |
| 8/9/19 | 1-Overnight | 1 | 8/24/19 | 4-Dinner | 1 |
| 8/9/19 | 2-Breakfast | 1 | 8/24/19 | 1-Overnight | 4 |
| 8/9/19 | 3-Lunch | 1 | 8/24/19 | 2-Breakfast | 4 |
| 8/9/19 | 4-Dinner | 1 | 8/24/19 | 3-Lunch | 4 |
| 8/9/19 | 3-Lunch | 2 | 8/25/19 | 1-Overnight | 1 |
| 8/9/19 | 4-Dinner | 2 | 8/25/19 | 2-Breakfast | 1 |
| 8/9/19 | 1-Overnight | 3 | 8/26/19 | 4-Dinner | 5 |
| 8/9/19 | 2-Breakfast | 3 | 8/26/19 | 2-Breakfast | 6 |
| 8/9/19 | 4-Dinner | 3 | 8/26/19 | 3-Lunch | 6 |
| 8/9/19 | 1-Overnight | 4 | 8/26/19 | 4-Dinner | 6 |
| 8/9/19 | 2-Breakfast | 4 | 8/27/19 | 1-Overnight | 5 |
| 8/9/19 | 4-Dinner | 4 | 8/27/19 | 1-Overnight | 6 |
| 8/9/19 | 1-Overnight | 5 | 8/27/19 | 2-Breakfast | 6 |
| 8/9/19 | 2-Breakfast | 5 | | | |
| 8/9/19 | 3-Lunch | 5 | | | |
| 8/9/19 | 1-Overnight | 6 | | | |
| 8/9/19 | 2-Breakfast | 6 | | | |
| 8/9/19 | 3-Lunch | 6 | | | |
| 8/10/19 | 2-Breakfast | 2 | | | |
| 8/10/19 | 3-Lunch | 2 | | | |
| 8/10/19 | 4-Dinner | 2 | | | |
| 8/10/19 | 1-Overnight | 4 | | | |
| 8/10/19 | 2-Breakfast | 4 | | | |
| 8/10/19 | 3-Lunch | 4 | | | |
| 8/15/19 | 1-Overnight | 5 | | | |
| 8/15/19 | 2-Breakfast | 5 | | | |
| 8/15/19 | 3-Lunch | 5 | | | |
| 8/15/19 | 4-Dinner | 5 | | | |
| 8/16/19 | 1-Overnight | 1 | | | |
| 8/16/19 | 3-Lunch | 1 | | | |
| 8/16/19 | 4-Dinner | 1 | | | |
| 8/16/19 | 1-Overnight | 5 | | | |
| 8/17/19 | 2-Breakfast | 1 | | | |
| 8/17/19 | 3-Lunch | 1 | | | |
| 8/17/19 | 4-Dinner | 1 | | | |

Reasons that monitoring periods were not completed included: loss of signal from CGM, detachment of CGM, or detachment of infusion set during monitoring period.

From 8/11/19-8/15/19 pigs were switched to AndroidAPS with a novel insulin formulation, and thus results from that period were not included in this study.

From 8/21/19-8/24/19 pigs #2, #3, #5, #6 were participating in a pharmacokinetic experiment for a novel insulin formulation not included in this study, thus were not available for closed-loop testing.

Once Loop studies began, a maximum of 3 pigs could be set-up on Loop per day due to rig interference. Rig interference only occurred during Loop experiments and was solved by reducing the number of pigs to three.

**Figure S2. Repeat measures (completed observation periods) for each pig by meal and algorithm.**

| Pig | Algorithm | Breakfast | Lunch | Dinner | Overnight | Total |
|---|---|---|---|---|---|---|
| 1 | AndroidAPS | 4 | 4 | 4 | 4 | **16** |
| | Loop | 4 | 3 | 3 | 3 | **13** |
| 2 | AndroidAPS | 2 | 3 | 3 | 1 | **9** |
| | Loop | 2 | 2 | 2 | 1 | **7** |
| 3 | AndroidAPS | 2 | 1 | 3 | 2 | **8** |
| | Loop | 2 | 1 | 0 | 1 | **4** |
| 4 | AndroidAPS | 3 | 3 | 3 | 3 | **12** |
| | Loop | 3 | 3 | 2 | 2 | **10** |
| 5 | AndroidAPS | 4 | 3 | 3 | 4 | **14** |
| | Loop | 2 | 2 | 2 | 2 | **8** |
| 6 | AndroidAPS | 3 | 3 | 2 | 2 | **10** |
| | Loop | 2 | 1 | 1 | 1 | **5** |
| **All** | **AndroidAPS** | **18** | **17** | **18** | **16** | **69** |
| | **Loop** | **15** | **12** | **10** | **10** | **47** |

* The values in this table represent the number repeated observations for each experimental condition (Number of times each pig was tested for a specific meal and algorithm combination). They are not independent measurements, but instead help to explain the within-subject variation for each condition (meal and algorithm). This is accounted for in analysis with a restricted maximum likelihood repeated measures mixed model which is used in the text for comparisons. Ultimately there are six subjects (pigs) who have measurements for 8 different conditions (2 algorithms, 4 meals). This results in a maximum of 48 independent measurements for the study. The only subject/condition combination that is not represented is Pig 3 at Dinner on Loop.

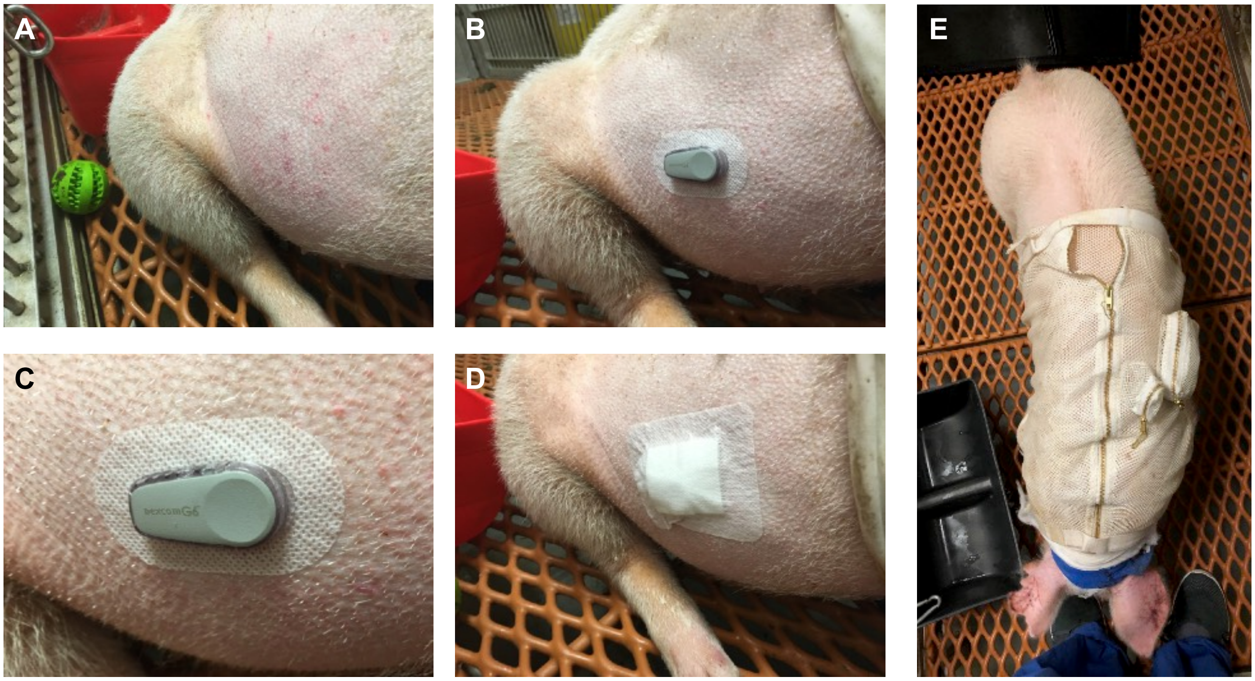


**Figure S1. Application of glucose sensors. (A)** Before application of the Dexcom G6 CGM the pig's side was shaved and then washed with chlorhexidine, rinsed with water and followed by 70% isopropyl alcohol. **(B,C)** Dexcom G6 sensor was applied and edges were smoothed down. **(D)** Skin-tac was lightly applied on the skin surrounding the sensor and allowed to dry. A Primapore pad was applied overtop the sensor to protect it from scratching or being bumped. **(E)** Overhead image of pig in jacket with the insulin pump in the jacket

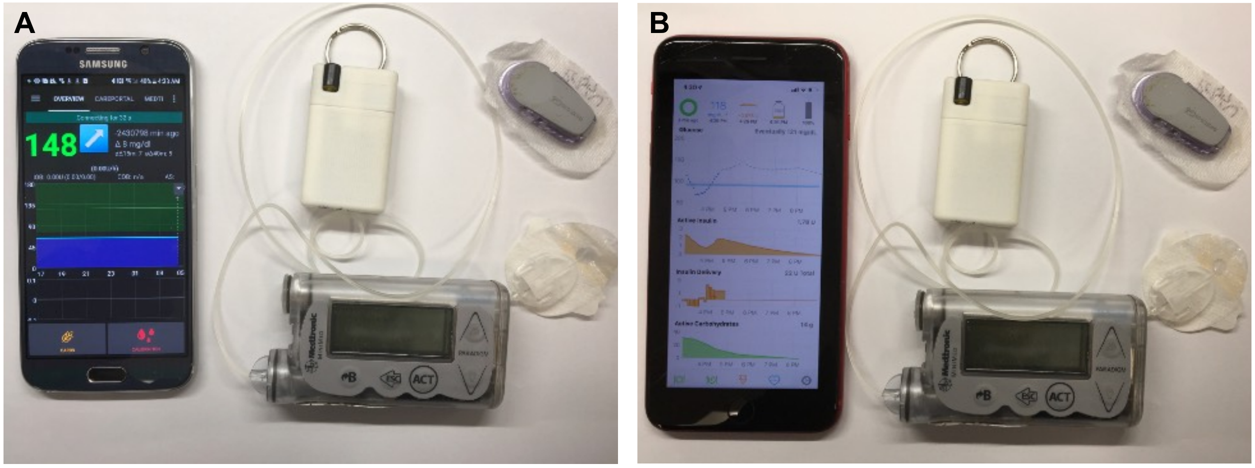


**Figure S2. Closed-loop devices.** Closed-loop set up including compatible Medtronic pump, Medtronic MiniMed Silhouette infusion set, RileyLink, and Dexcom G6 sensor and **(A)** Android phone running AndroidAPS or **(B)** iPhone running Loop.

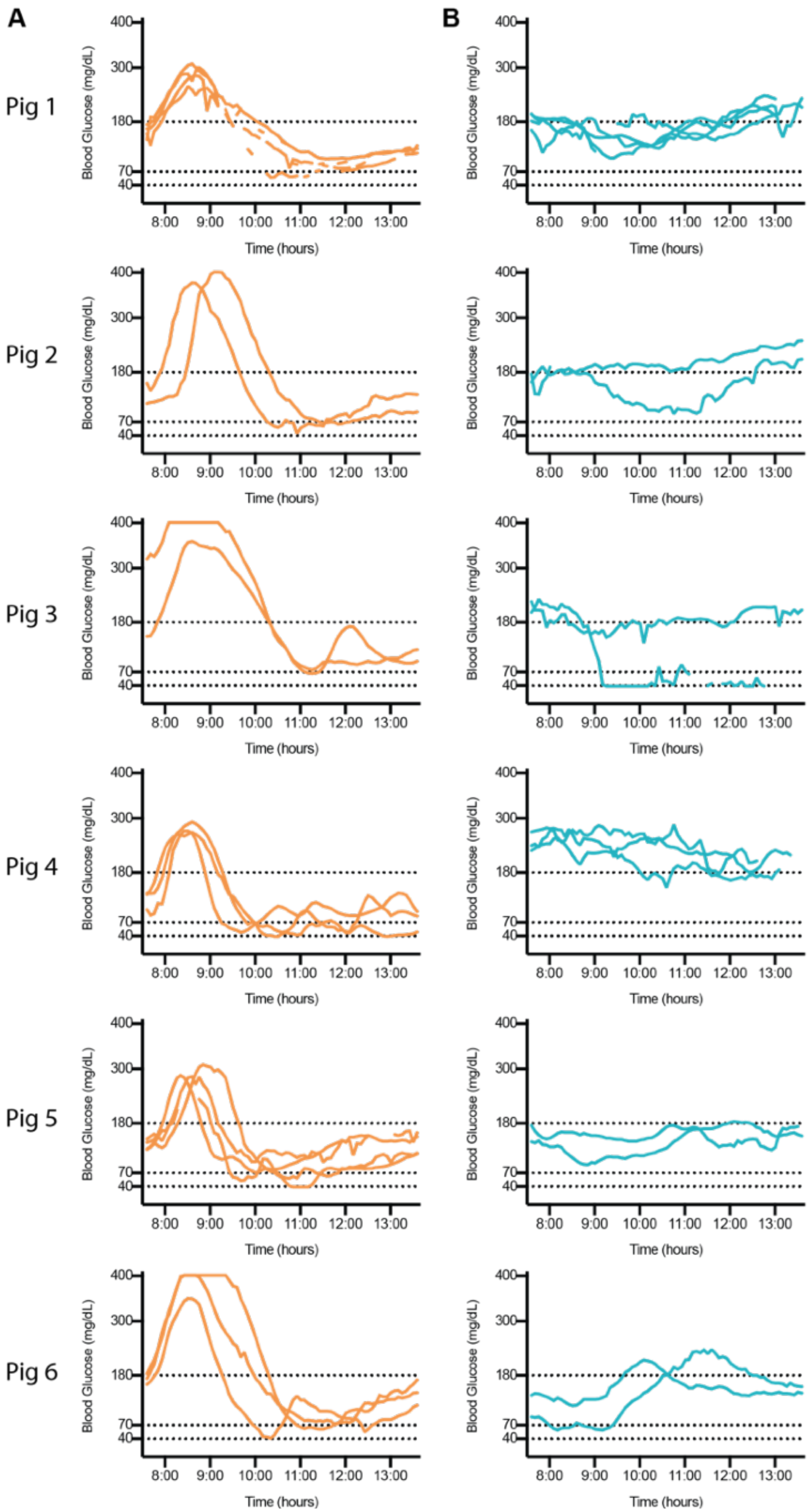


**Figure S3. Breakfast glucose curves for individual pigs.** Glucose traces for individual pigs for breakfast (66g simple carbs) on **(A)** AAPS or **(B)** Loop algorithms. Time in range (TIR) was defined as time where CGM measured glucose between 70-180 mg/dL and hypoglycemia was defined <70 mg/dL. Glucose curves were shifted on the x-axis to align the meal start times.

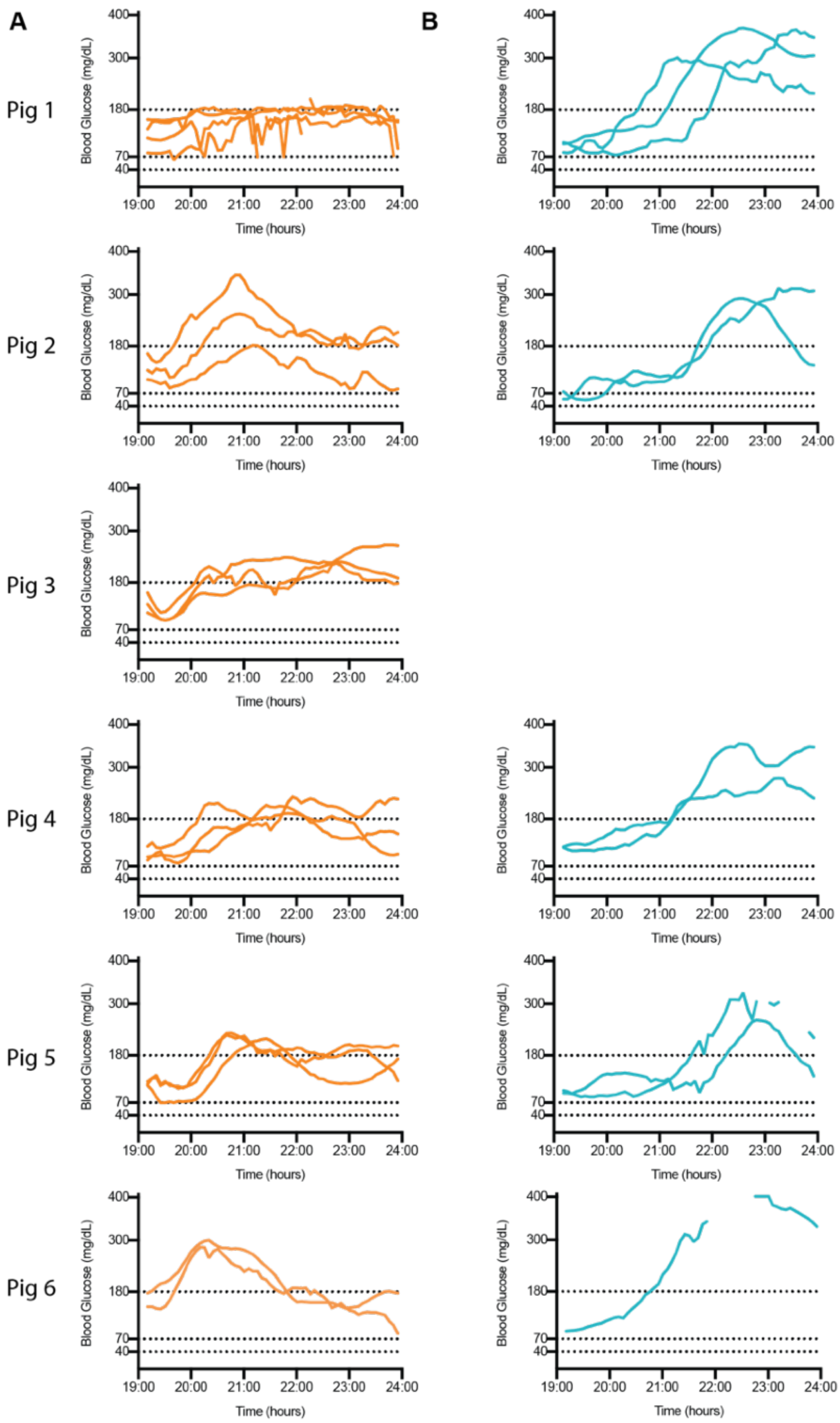


**Figure S5. Dinner glucose curves for individual pigs.** Glucose traces for individual pigs for dinner (143g mixed meal) on **(A)** AAPS or **(B)** Loop algorithms. Time in range (TIR) was defined as time where CGM measured glucose between 70-180 mg/dL and hypoglycemia was defined <70 mg/dL. Glucose curves were shifted on the x-axis to align the meal start times.

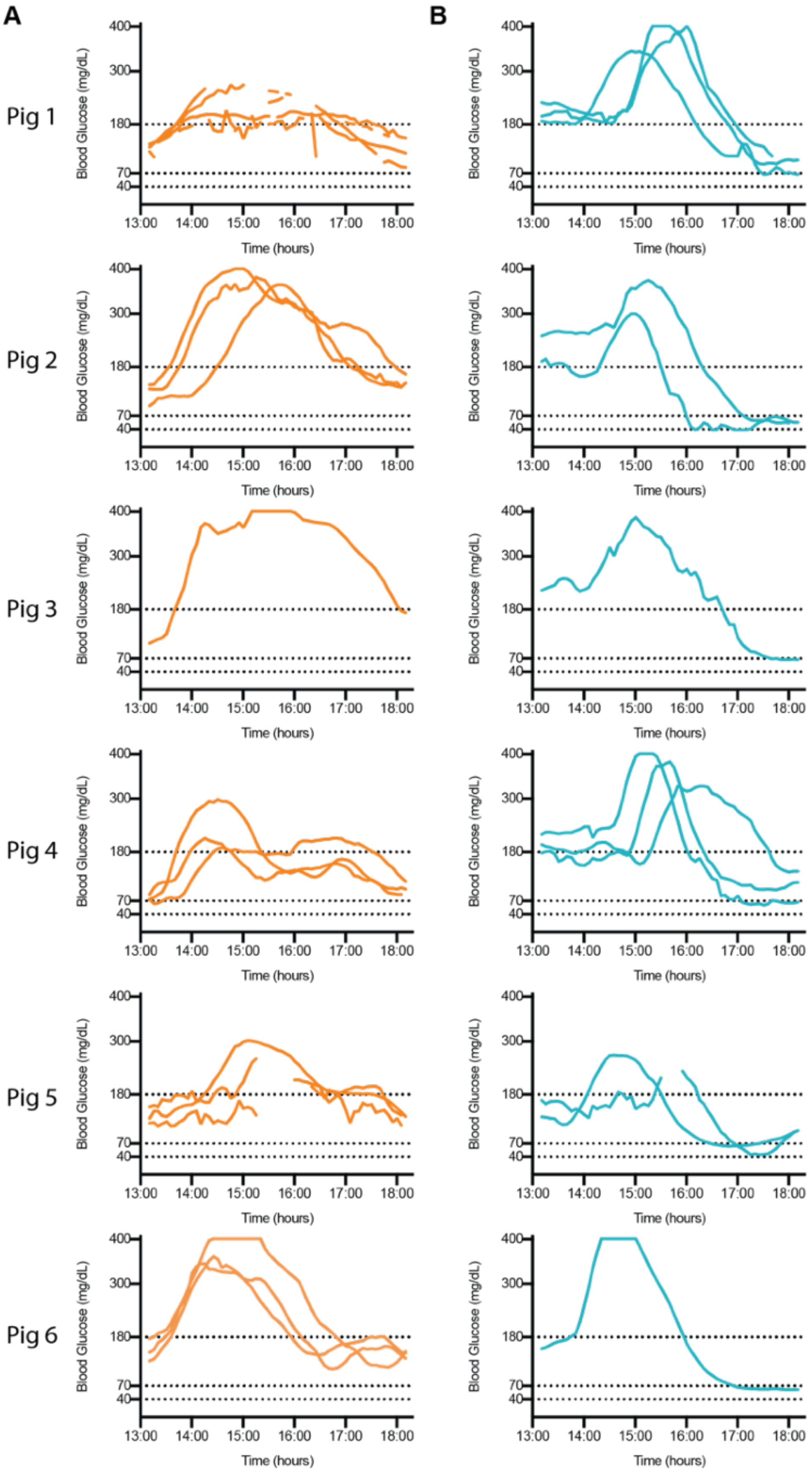


**Figure S4. Lunch glucose curves for individual pigs.** Glucose traces for individual pigs for lunch (143g mixed meal) on **(A)** AAPS or **(B)** Loop algorithms. Time in range (TIR) was defined as time where CGM measured glucose between 70-180 mg/dL and hypoglycemia was defined <70 mg/dL. Glucose curves were shifted on the x-axis to align the meal start times.

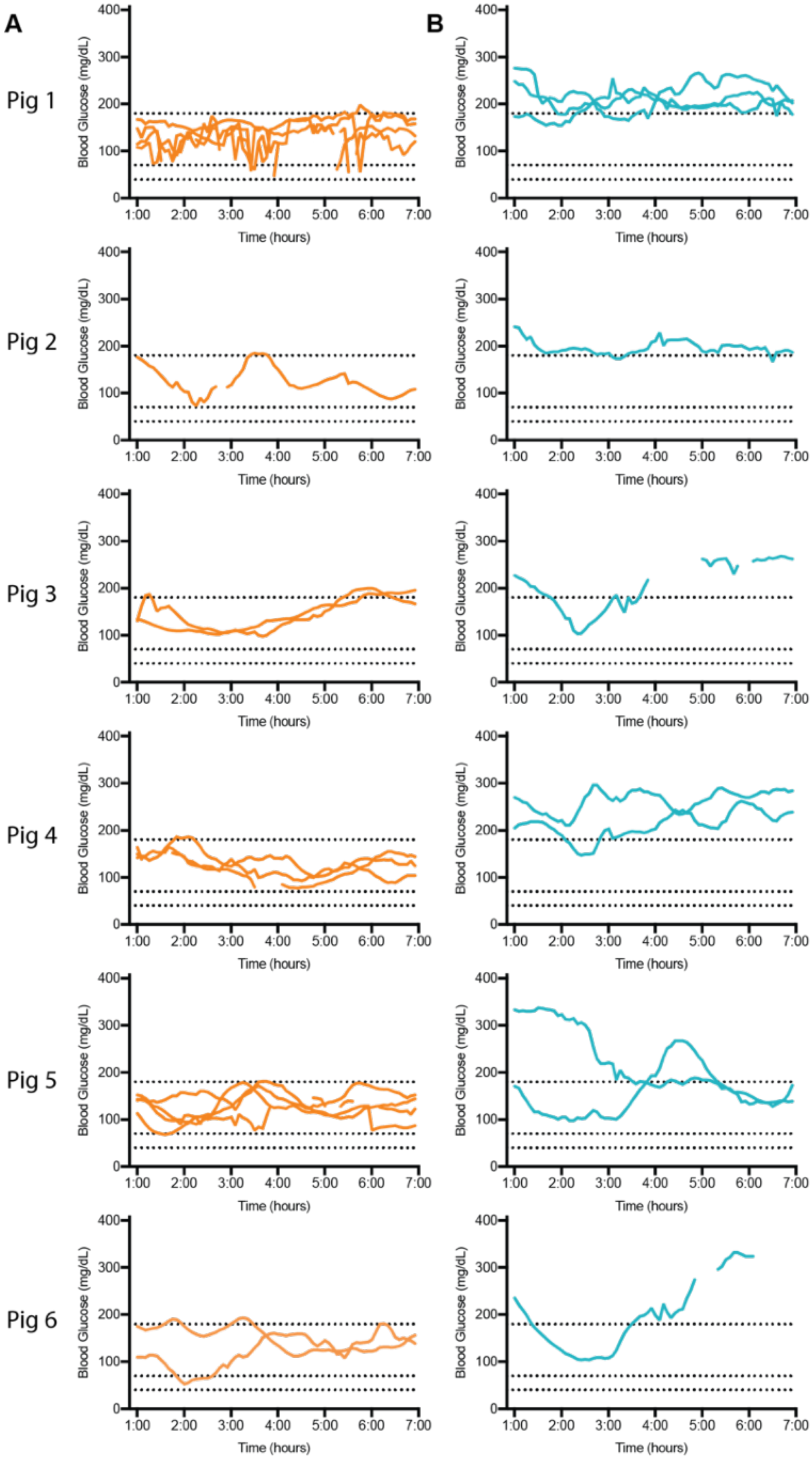


**Figure S6. Overnight glucose curves for individual pigs.** Glucose traces for individual pigs for overnight (no meal) on **(A)** AAPS or **(B)** Loop algorithms. Time in range (TIR) was defined as time where CGM measured glucose between 70-180 mg/dL and hypoglycemia was defined <70 mg/dL. Glucose curves were shifted on the x-axis to align the start times.

**Table S3. Percent time in range (70-180 mg/dL) and time in hypoglycemia**

| | | Breakfast | Lunch | Dinner | Overnight |
|---|---|---|---|---|---|
| **AAPS** | **% Time CGM <70 mg/dL** | 6 +2/-1 | 1.4 +0.4/-0.3 | 1.6 +0.5/-0.4 | 2.1 +0.7/-0.5 |
| | **% Time CGM 70-180 mg/dL** | 54 ± 7 | 41 ± 7 | 52 ± 7 | 90 ± 7 |
| **Loop** | **% Time CGM <70 mg/dL** | 2.2 +0.7/-0.5 | 9 +3/-3 | 2.1 +0.8/-0.6 | 1.4 +0.5/-0.4 |
| | **% Time CGM 70-180 mg/dL** | 50 ± 7 | 31 ± 7 | 42 ± 8 | 22 ± 8 |

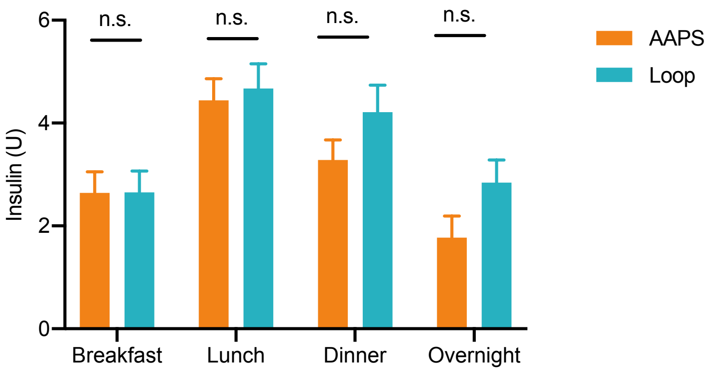


**Figure S7. Insulin delivery by meal.** Total insulin delivered during the 5 hours following the beginning of each monitoring period. Data shown as least squares mean ± standard error.

## REFERENCES

1. Renner S, Dobenecker B, Blutke A, et al. Comparative aspects of rodent and nonrodent animal models for mechanistic and translational diabetes research. *Theriogenology.* 2016;86(1879-3231 (Electronic)):406-421.

2. Larsen MO, Rolin B. Use of the Göttingen minipig as a model of diabetes, with special focus on type 1 diabetes research. *ILAR J.* 2004;45(3):303-313.

3. Tang H, Mayersohn M. Porcine Prediction of Pharmacokinetic Parameters in People: A Pig in a Poke? *Drug Metab Dispos.* 2018;46(11):1712-1724.

4. Davis SS, Illum L, Hinchcliffe M. Gastrointestinal transit of dosage forms in the pig. *J. Pharm. Pharmacol.* 2001;53(1):33-39.

## GRAPHICAL HEADLIGHTS

- Open-source automated insulin delivery systems AndroidAPS and Loop were compared in diabetic pigs without meal announcements
- Faster pharmacokinetics in pigs presents a unique opportunity to observe algorithm performance simulating use with a next-generation ultrafast insulin formulations
- AndroidAPS had a greater time in the target glucose range than Loop during the overnight period (87% vs. 22%)
- Loop's overnight performance was affected by poor management of the dinner mealtime glucose load
